# Is it bowel-associated dermatosis-arthritis syndrome induced by small intestinal bacteria overgrowth?

**DOI:** 10.1186/s40064-016-3236-8

**Published:** 2016-09-13

**Authors:** Hongjun Zhao, Lijuan Zhao, Wei Shi, Hui Luo, Liping Duan, Yunhui You, Yisha Li, Xiaoxia Zuo

**Affiliations:** 1 Department of Rheumatology and Immunology, Xiangya Hospital, Central South University, 87 Xiangya Road, Changsha, Hunan Province 410008 People’s Republic of China; 2 Departments of Dermatology, Xiangya Hospital, Central South University, Changsha, Hunan Province 410008 People’s Republic of China

**Keywords:** Dermatosis, Arthritis, Inflammatory bowel disease

## Abstract

**Introduction:**

Bowel-associated dermatosis-arthritis syndrome is rare systemic diseases characterized by a prodrome of fever, chills, and influenza-like symptoms with subsequent skin eruptions, myalgias, and polyarthralgias. It is reported to be occurred in Intestinal bypass surgery and inflammatory bowel disease.

**Case description:**

Herein, we described a 29-years-old man with Bowel-associated dermatosis-arthritis syndrome. He had no history of gastrointestinal surgery and inflammatory bowel disease. Distribution of the gut bacterial flora showed small intestinal bacterial overgrowth.

**Discussion and Evaluation:**

It is rarely form as Non-intestinal bypass road and non-inflammatory bowel disease was induced by small intestinal bacteria overgrowth.

**Conclusions:**

We concluded that Immuno-inflammatory response to overgrowth of intestinal bacterial antigen induce the clinical symptoms of bowel-associated dermatosis-arthritis syndrome.

## Background

Bowel-associated dermatosis-arthritis syndrome (BADAS) was first described by Ely in 1980s with characteristics of fever, chills, and influenza-like symptoms with subsequent skin eruptions, myalgias, and polyarthralgias (Ely PH.1980). It is called as short-bowel syndrome since the occurrence rate among patients of jejuno-ileum bypass bariatric surgery is over 20 %. BADAS is reported to be occurred in ileoanal anastomosis, biliopancreatic diversion as well as Billroth II gastrectomy. Jorizzo described four cases (Jorizzo et al. 1983), these patients had the same clinical manifestations with short-bowel syndrome, however, they had never received any operations which mentioned above. The only common point of these patients was that they all had gastrointestinal disease and similar cases had been continuously reported afterwards (Delaney et al. 1989; Kemp and Gin 1990; Geary et al. 1999; Cox and Palmer 2003). Scholars believed that it was a complication of inflammatory bowel disease and put forward the concept of bowel-associated dermatosis-arthritis syndrome (BADAS) to cover the non-intestinal bypass short-bowel syndrome. A number of literatures reported that SIBO was involved in the pathogenesis of the disease. Short-bowel syndrome predisposes the patient to Small Intestine Bacterial Overgrowth (SIBO) (Goulet and Joly 2010). It has been shown that patients with Crohn’s disease (CD) have a higher risk of SIBO development (Greco et al. 2015). Herein, we described a patient with BADAS induce by small intestinal bacterial overgrowth.

## Case presentation

**Fig. 1:**
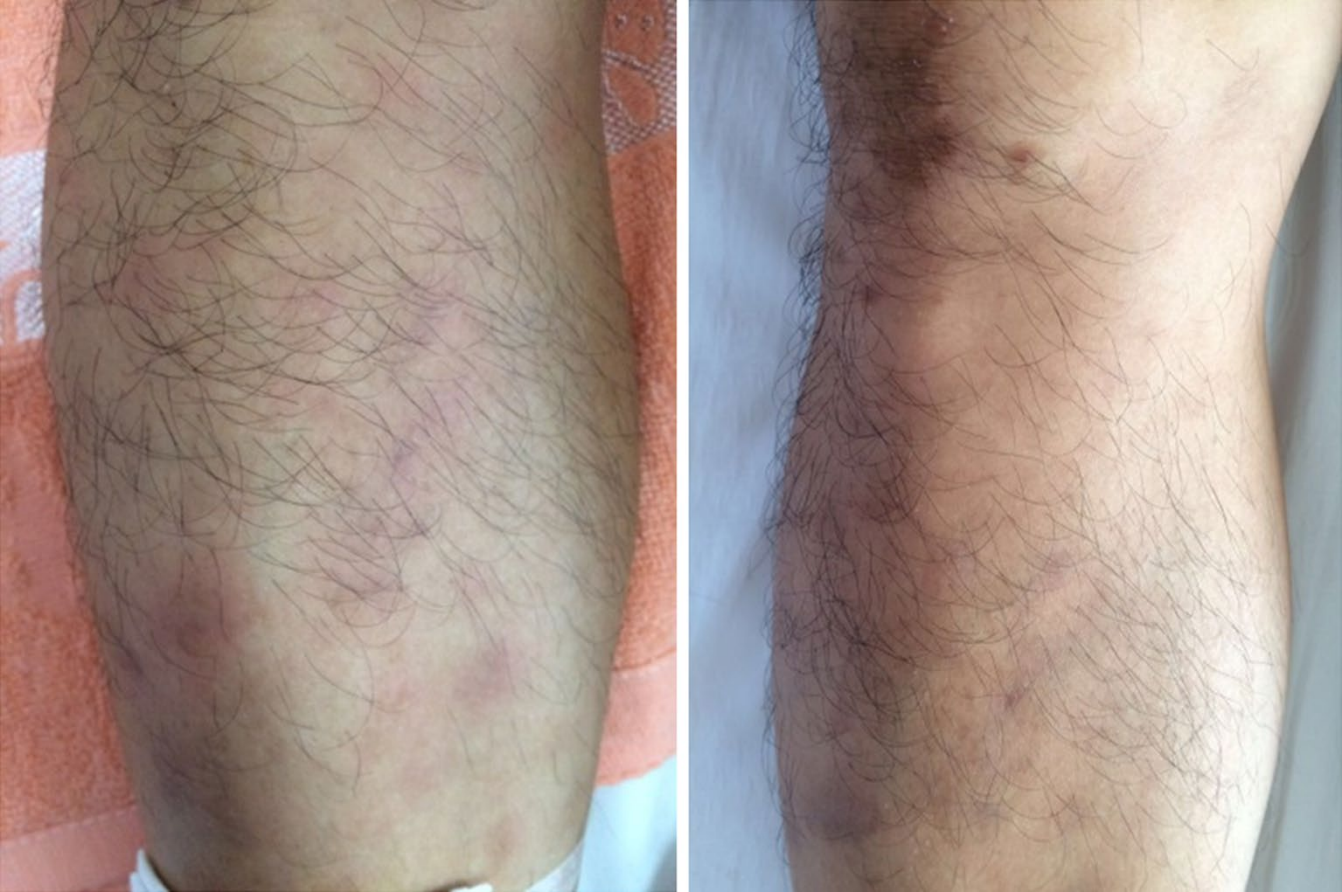
The picture of red tubercular rashes on both lower limbs. *Note*: The patient has red tubercular rashes on both lower limbs,erythematous nodular rashes with diameter of about 3-10 mm and vague boundaries on his limbs

**Fig. 2:**
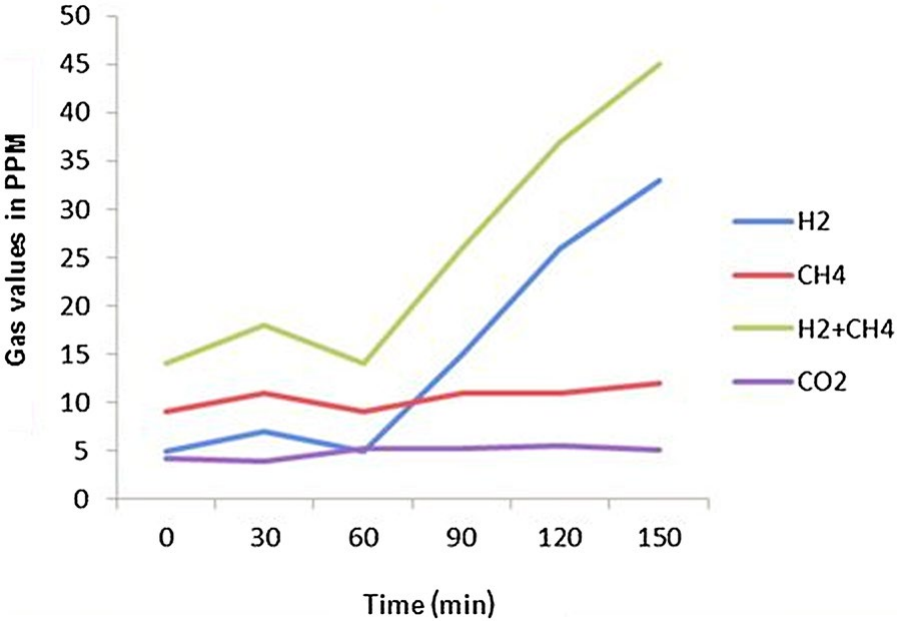
The result of small intestinal bacterial overgrowth (SIBO). *Note*: The result of small intestinal bacterial overgrowth (SIBO) test is positive. Hydrogen-methane breath test with glucose as the substrate, after taking glucose for 1 h, the concentration of H2 reached the peak value which implied the bacterial overgrowth in the ileum

**Fig. 3:**
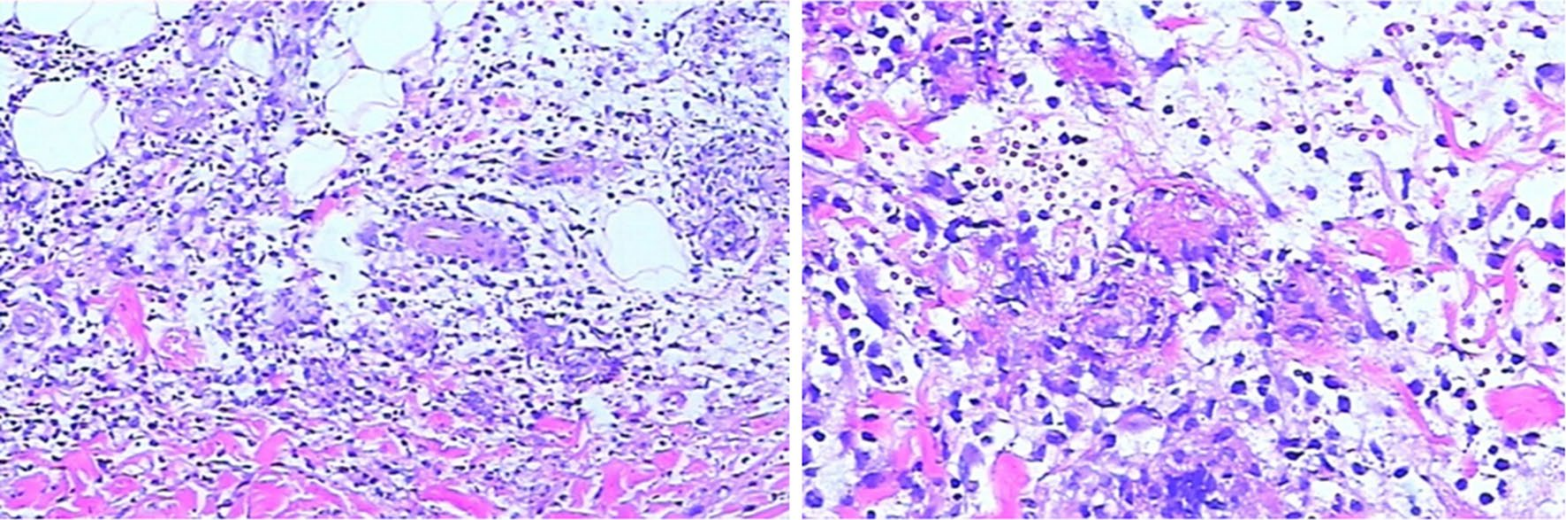
The dermal pathology at rashes. *Note*: The dermal pathology at rashes manifests lymphocyte and neutrophile granulocyte around the deep blood vessels of dermis (rash skin of left limb, HE staining, original magnification 200–400)

A 29-year-old man complained of joint pain, fever, rash and intermittent diarrhea for about a year. One year ago, he had arthralgia and swelling in the ankles, knees, wrists and digital joints, together with fever (>38.7 °C) and erythematous nodular rashes with diameter of about 1 cm turn up on his limbs,These rashes have vague boundaries with the normal skin nearby (Fig. 1). The watery and soft stools occurred intermittently, with 3–4 bouts a day and 4–5 days a month. He had been diagnosed with reactive arthritis. After taking non-steroidal anti-inflammatory drugs (NSAIDs) such as celecoxib, arthritis, fever and rash were relieved for a short time before recurrence. He didn’t undergo gastrointestinal surgery. After being admitted to our hospital, his blood cell count, urinary analysis and stool routine with occult blood test were normal. The erythrocyte sedimentation rate (ESR) 104 mm/h (0–21 mm/h), C-reactive protein (CRP) 94.7 mg/L (0–8 mg/l), interleukin-6(IL-6) 123.8 ng/ml (0–7 ng/ml) were all increased. HLA-B27 was negative. No abnormalities were found in the procalcitonin, blood culture, Multiple virus antibody, T-SPOT.TB, tumor marker, anti-nuclear antibody (ANA), anti-dsDNA antibody (AdsDNA), anti-ENA antibodies, rheumatoid factor (RF), anti-CCP, and anti-neutrophil cytoplasmic antibodies (ANCA). Electronic gastrointestinal endoscope (FUJINON EVE 400 SERIES VIDEO SYSTEM), results of abdomen CT and bone marrow aspiration cytology were normal. Distribution of the gut bacterial flora showed dysbacteriosis with 10 % klebsiellapneumoniae, 80 % *E. coli* and 10 % peptostreptococcus. The result of SIBO test (hydrogen-methane breath test with lactulose as the substrate) was positive (Fig. 2). A further skin biopsy was taken from a rash on the left lower extremity and Low power microscope shows lymphocyte and neutrophile granulocyte around deep blood vessels of dermis (Fig. 3). He was diagnosed with BADAS (caused by small intestinal bacterial overgrowth). The patient was improved dramatically after treatment with doxycycline 0.2 g/day and methylprednisolone 40 mg/day for a week. Glucocorticoid tapered gradually to withdrawal. The patient was followed up for 12 months without recurrent symptoms.

## Discussion and evaluation

BADAS is a non-infectious neutrophilic dermatosis with prominent of features of rashes and joint pain. Typical rashes are red spots with diameter of 3–10 mm and vague boundaries. The rashes gradually harden in the following 1 or 2 days and turn to papulopustule with diameter of 2–4 mm. The process of this disease could last for 2–8 days and showed as a self-limited process (Patton et al. 2009; Slater et al. 2004; Kawakami et al. 2006). The rashes are mainly distributed on the body and limbs (Jorizzo et al. 1983), and they may be featured by pruritus, pain or no subjective symptom (Slater et al. 2004). Polyarthritis may be involved in patients which had peripheral joints like IP joints and wrist joints, however, but there is no imageological change, joint destruction or deformity (Utsinger 1980). Laboratory indexes such as RF, immune globulin and uric acid are all within normal limits, however, some patients may have elevated cryoglobulin (Patton et al. 2009). In microbiological examination, both of blood culture and rash fester culture results are negative (Truchuelo et al. 2013). The dermal pathology of BADAS is usually the same as acute febrile neutrophilic dermatosis (SWEET) syndrome. It mainly shows infiltration of mature neutrophile granulocyte in dermis and sometimes edema of dermal papilla. It had no fibrinoid necrosis or blood vessel infarct, which is the main difference from leukocytoclastic vasculitis (Patton et al. 2009; Ashok and Kiely 2007). However, other research showed that the dermal pathology changes with the development of the disease is not an essential condition for diagnosing the BADAS (Patton et al. 2009). In the course of clinical diagnosis and treatment, BADAS also must be distinguished from extra-intestinal manifestations of inflammatory bowel disease (Adams and Eksteen 2006; Trikudanathan et al. 2012; Marineaţă et al. 2014; Brown et al. 2015).

As the cause is not clear, it is now generally accepted that it is an abnormal immune response of residual intestine after intestinal survey or abnormal intestine of inflammatory bowel disease to the bacterial overgrowth. SIBO is defined as nonpathogenic bacteria increase over 10^5^cfuin 1 ml of small intestine content. Due to the lack of specific symptoms, SIBO is often misdiagnosed. In fact, SIBO occurrence is fairly frequent. SIBO might present in more than 60 % patients with abdominal pain (Siniewicz-Luzenczyk et al. 2015). In systemic sclerosis, 38 % patientswith intestinal complaints were diagnosed with SIBO (Tauber et al. 2014). The formation of immune complex, access to blood and deposition in tissues and organs are crucial in the occurrence of disease which could induce various related clinical symptoms (Slater et al. 2004; Utsinger 1980; Dicken 1986; Jorizzo et al. 1984) and this theory can be indirectly proved by the curative effect of antibiotics and glucocorticoid to the disease. Glucocorticoid is as the main medicine for this disease and antibiotics (metronidazole, tetracycline and sulfonamides) are also used with different effects. Resuming the normal anatomy of intestine or curing potential gastrointestinal disease also has certain mitigative effect (Ashok and Kiely 2007).

About 20 % of patients of jejunoileum bypass bariatric surgery are likely to have the symptoms of rash or joint pain (Ely 1980). Other non-bypass surgery or disease of digestive tract such as inflammatory bowel disease has had already been continuously reported, which is secondary to the BADAS (Cox and Palmer 2003; Patton et al. 2009). In our case, the clinical manifestation and dermal pathology of rashes are the same with those diseases mentioned above, however, there is no intestinal surgery, nor any evidence of inflammatory bowel disease. Only the hydrogen-methane breath test with lactulose as the substrate indicates SIBO. The disease may not merely occur among patients of jejuno-ileum bypass bariatric surgery and inflammatory bowel disease as mentioned in previous literature. Immuno-inflammatory response to bacterial antigen may be caused by any factors which caused the overgrowth of intestinal bacteria, mediate formation of immune complex and hence induce the clinical symptoms of BADAS. Therefore, whether the patients had specific digestive system surgery or medical history, as organic or functional factor which may cause intestinal bacterial overgrowth should be taken into consideration during the clinical diagnosis of physician. Patient with only SIBO, non-intestinal bypass road and non-inflammatory bowel disease could also be diagnosed as BADAS.

## Conclusion

In clinical practice, rheumatologists can easily recognize BADAS patients with jejunoileum bypass bariatric surgery or inflammatory bowel disease, and more attention should be paid to bacteria translocation, flora imbalance and SIBO which may also result in BADAS. Correctly understanding the factors that influence the occurrence of BADAS will help us getting to understand more about diagnosing this relatively rare disease.

## Abbreviations

BADAS: bowel-associated dermatosis-arthritis syndrome
SIBO: small intestinal bacterial overgrowth
CD: Crohn’s disease
NSAIDs: non-steroidal anti-inflammatory drugs
ESR: erythrocyte sedimentation rate
CRP: C-reactive protein
IL-6: interleukin-6
HLA-B27: human leukocyte antigen B27
T-SPOT.TB: T-cell spot of TB test
AdsDNA: anti-dsDNA antibody
anti-ENA antibodies: anti-extractable nuclear antigen antibodies
RF: rheumatoid factor
anti-CCP: anti-cyclic peptide containing citrulline
ANCA: anti-neutrophil cytoplasmic antibodies
SWEET syndrome: acute febrile neutrophilic dermatosis syndrome
ANA: anti-nuclear antibody
IP: interphalangeal
